# Tau exon 10 alternative splicing and tauopathies

**DOI:** 10.1186/1750-1326-3-8

**Published:** 2008-07-10

**Authors:** Fei Liu, Cheng-Xin Gong

**Affiliations:** 1 Department of Neurochemistry, New York State Institute for Basic Research in Developmental Disabilities, Staten Island, New York 10314, USA; 2 Jiangsu Key Laboratory of Neuroregeneration, Nantong University, Nantong, Jiangsu 226001, PR China

## Abstract

Abnormalities of microtubule-associated protein tau play a central role in neurofibrillary degeneration in several neurodegenerative disorders that collectively called tauopathies. Six isoforms of tau are expressed in adult human brain, which result from alternative splicing of pre-mRNA generated from a single *tau *gene. Alternative splicing of *tau *exon 10 results in tau isoforms containing either three or four microtubule-binding repeats (3R-tau and 4R-tau, respectively). Approximately equal levels of 3R-tau and 4R-tau are expressed in normal adult human brain, but the 3R-tau/4R-tau ratio is altered in the brains in several tauopathies. Discovery of silence mutations and intronic mutations of *tau *gene in some individuals with frontotemporal dementia with Parkinsonism linked to chromosome 17 (FTDP-17), which only disrupt tau exon 10 splicing but do not alter tau's primary sequence, demonstrates that dysregulation of tau exon 10 alternative splicing and consequently of 3R-tau/4R-tau balance is sufficient to cause neurodegeneration and dementia. Here, we review the gene structure, transcripts and protein isoforms of tau, followed by the regulation of exon 10 splicing that determines the expression of 3R-tau or 4R-tau. Finally, dysregulation of exon 10 splicing of *tau *in several tauopathies is discussed. Understanding the molecular mechanisms by which *tau *exon 10 splicing is regulated and how it is disrupted in tauopathies will provide new insight into the mechanisms of these tauopathies and help identify new therapeutic targets to treat these disorders.

## Introduction

Tau is a microtubule-associated protein expressed predominantly in the neuron. Its major known biological function is to stimulate microtubule (MT) assembly and to stabilize MT network. Thus, tau plays important roles in morphogenesis, axonal extension, as well as axonal vesicle and protein transport in neurons. The biological function of tau is regulated by the degree of its phosphorylation. Since the discovery that abnormally hyperphosphorylated tau makes up paired helical filaments (PHFs) and straight filaments of neurofibrillary tangles (NFTs) in brains of individuals with Alzheimer disease (AD) [1,2], tau and the role of its abnormalities in neurodegeneration have been a hot subject of research. In addition to AD, aggregation of hyperphosphorylated tau in the brain is also seen in several other neurodegenerative diseases, such as progressive supranuclear palsy (PSP), corticobasal degeneration (CBD), frontotemporal dementia with Parkinsonism linked to chromosome 17 (FTDP-17), Pick's disease (PiD), Down syndrome (DS), postencephalitic Parkinsonism, and Niemann-Pick disease. This diverse set of sporadic and familial neurodegenerative disorders are called collectively as "tauopathies" [3,4].

Adult human brain expresses six isoforms of tau protein, which are derived from a single *tau *gene as a result of alternative splicing of its pre-mRNA [5]. The six tau isoforms differ from each other by the presence or absence of one or two inserts (29 or 58 amino acids) in the N-terminal part and by the presence of either three or four MT-binding repeats (R) in the C-terminal half. The presence or absence of the second MT-binding repeats is resulted from alternative splicing of exon 10 of the *tau *gene, leading to the expression of either 4R-tau or 3R-tau [6,7]. Normal adult human brain expresses approximately equal levels of 3R-tau and 4R-tau [8,9]. Altered 3R/4R-tau ratios have been observed in several tauopathies [10-12]. In some families of FTDP-17, alterations of exon 10 splicing of *tau *due to silence or intronic mutations lead to the disease [10]. These observations indicate that dysregulation of *tau *exon 10 splicing can cause or contribute to neurodegeneration.

In this article, we first briefly describe the gene structure, transcripts and protein isoforms of tau. Then, we review the regulation of exon 10 splicing that determines the expression of 3R-tau or 4R-tau. Finally, dysregulation of exon 10 splicing of *tau *in several tauopathies is discussed.

## Gene structure, transcripts and proteins of *tau*

**Figure 1:**
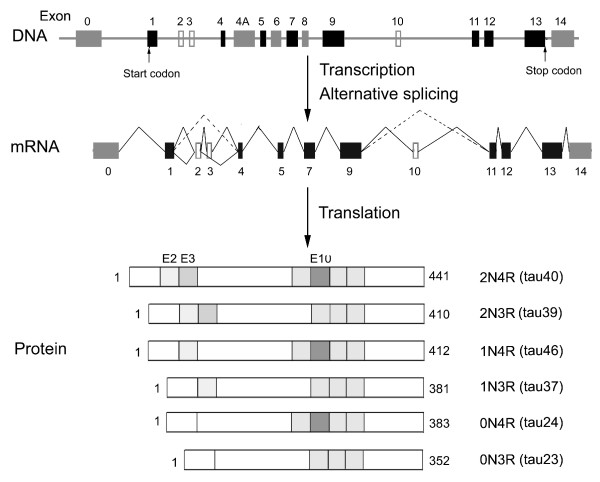
**The gene, mRNA and protein isoforms of tau**. In tau genomic structure (top panel), the black boxes represent constitutive exons, and the gray and empty boxes represent alternative spliced exons. The middle panel demonstrates mRNAs of tau in adult human brain. A total six mRNAs are generated by alternative splicing of exons 2, 3 and 10, which is indicated by alternative lines linking these exons. The lower panel shows six isoforms of tau in adult human brain. Gray boxes represent the N-terminal inserts (coded by exons 2 and 3) or MT-binding repeats (coded by exons 9, 10, 11 and 12). The second MT-binding repeat coded by exon 10 is highlighted by dark gray box. The commonly used terms for each tau isoform are listed at the right side of the isoforms.

The single human tau gene is located over 100 kb on the long arm of chromosome 17 at band position 17q21.1, which contains 16 exons (Fig 1) [13,14]. Exons 1, 4, 5, 7, 9, 11, 12, and 13 are constitutive exons, and the remaining exons are subject to alternative splicing. Exon 1 is part of the promoter and is transcribed but not translated. Sequencing of the promoter region reveals a TATA-less sequence. The promoter region also contains consensus binding sites for transcription factors AP2, SP1, and GCF. The SP1-binding sites may control neuronal specific expression of tau [15,16]. Exons 4A, 6 and 8 are present in mRNA of the peripheral tissue and are never present in human brain. Exon 14 is part of the 3'untranslated region of tau mRNA [5,6]. Restriction analysis and sequencing show that *tau *gene contains two CpG islands, one associated with the promoter region and the other within exon 9.

The primary transcript of *tau *is processed to produce three different transcripts of 2, 6 and 9 kb, which are differentially expressed in the nervous system, depending upon stages of neuronal maturation and neuron types [5,6,17,18]. The MT-associated protein tau is produced from the 6-kb mRNA expressed primarily in neurons of the brain. The 2-kb *tau *mRNA produces a tau isoform that is localized to the nucleus [19], and the 9-kb transcript is restricted to the retina and the peripheral nervous system [18].

In the adult human brain, exons 2, 3 and 10 are alternatively spliced [14]. Exon 3 never appears independently of exon 2 [7]. Thus, the alternative splicing of these three exons yields to six combinations of mature mRNA and the corresponding six isoforms of tau protein (Fig. 1) [5]. The six tau isoforms differ from each other by the presence or absence of one or two inserts (29 or 58 amino acid residues, coded by exon 2 or exons 2 and 3) in the N-terminal part and the presence or absence of the second MT-binding repeat (encoded by exon 10) in the C-terminal portion. The apparent molecular weight of these tau isoforms ranges from 45 kDa to 65 kDa in SDS-PAGE. In the adult human brain, the ratio of 3R-tau and 4R-tau isoforms is ~1. On the other hand, tau isoforms with 2 inserts (2N), 1 insert (1N) and 0 insert (0N) in the N-terminal region comprise ~54%, ~37% and ~9%, respectively, of total tau [8,20]. Each of this isoforms appears to have some differential physiological roles since they are differentially expressed during development. In the fetal human brain, only the shortest tau isoform (exons 2, 3 and 10 are spliced out) is present [9]. In the peripheral nervous system, inclusion of exon 4a in the N-terminal half results in the expression of a higher molecular weight (~110 kDa) protein termed big tau [21,22].

The presence of many serine/threonine, proline, and arginine/lysine/histine residues in tau molecule bestows unusual characters with potential to be hyperphosphorylated, very poor secondary structure and basic protein, which linked to its biological function and pathologic changes in the diseases. The main biological functions of tau known are to stimulate MT assembly and to stabilize MT structure. Tau binds to MTs through its MT-binding repeats. 4R-tau isoforms are more efficient at promoting MT assembly and have a great MT-binding affinity than do 3R-tau isoforms [8] because the inter-repeat sequence between the first and second MT-binding repeats has more than twice the binding affinity of any other individual MT-binding repeats [23-26]. Therefore, tau from fetal brain promotes microtubule assembly less efficiently than tau from adult brain [27].

## Alternative splicing of *tau *exon 10

Alternative splicing of pre-mRNA, the differential inclusion or exclusion of portions of a nascent transcript into the final protein-coding mRNA, is widely recognized to be a ubiquitous mechanism for controlling protein expression. More than 60% of mammalian pre-mRNA is alternatively spliced, and this process is widely prevalent in the nervous system [28,29]. Splicing is catalyzed by the spliceosome, a macromolecular machine consisting of five small nuclear RNA (snRNA) molecules (U1, U2, U4, U5 and U6 snRNA) and as much as 150 proteins [30-32]. Each of the five snRNAs assembles with proteins to form small nuclear ribonucleoprotein particles (snRNP). A coordinated binding of the five snRNP to pre-mRNA results in the removal of each intron and the ligation of the flanking exons. Alternative splicing is controlled by multiple exonic and intronic *cis-*elements and *trans-*acting splicing factors. The element in an exon that increases inclusion of the alternatively spliced exon is called exonic splicing enhancer (ESE), and that decreases inclusion is called exonic splicing silencer (ESS). The element with similar function located in an intron is called intronic splicing enhancer (ISE) or intronic splicing silencer (ISS).

### Cis-elements in *tau *exon 10 and intron 10

**Figure 2:**
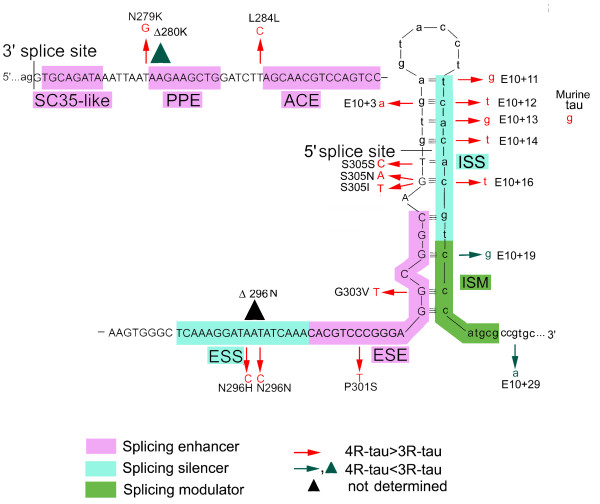
**Structure of exon 10 and intron 10 of *tau *gene**. Exon 10 is shown in capital letters and part of the franking intron 9 and intron 10 are shown in lowercase. The first half of exon 10 has three exonic splicing enhancers (SC35-like, PPE and ACE). A central exon splicing silencer (ESS) separates the 5' ESE elements from a less characterized ESE at the 3'-end of exon 10. Intron 10 elements include a bipartile intronic splicing silencer (ISS) and an adjacent intronic splicing modulator (ISM). In the interface between exon 10 and intron 10, there is a stem-loop structure. Mutations that cause an increase (red), decrease (dark green) or not yet known change (black) in the ratio of 4R/3R-tau are indicated. Triangles indicate deletion mutations.

Most alternative spliced exons contain one weak splice site. However, tau exon 10 has two weak splice sites, a weak 5' splice and a weak 3' splice site [33-35]. The exon is flanked by unusually large intron 9 (13.6 kb) and intron 10 (3.8 kb). These features of *tau *exon 10 lead to much complicated regulation. Several short *cis*-elements in exon 10 and intron 10, which modulate the use of the weak 5' and 3' splice sites, have been identified and extensively characterized [10,36]. The 5' end of exon 10 contains three ESEs: a SC35-like enhancer, a polypurine enhancer (PPE), and an A/C-rich enhancer (ACE) (Fig. 2). Following the ESEs region, there is an exon splicing silencer (ESS). In addition, the 3' end of exon 10 contains another ESE sequence between the ESS and the 5' splice site. In intron 10, there are bipartite elements composed of the ISS (E10+11 to E10+18) and the intronic splicing modulator (ISM) (E10+19 to E10+26). Deletion assay revealed opposite effects of the ISS and ISM on E10 splicing [35]. The ISM is not an enhancer by itself, but functions only in the presence of the ISS and counteracts ISS-mediated inhibition of the 5' splice site. Mutation in these elements may disrupt their function in alternative splicing of exon 10. A total of 14 mutations within the six elements (PPE, ACE, ESS, ESE, ISS and ISM) have now been identified in individuals with tauopathies. These mutations include N279K and Δ280 in PPE; L284L in ACE; N296H, N296N and Δ296N in ESS; P301S G303V in ESE; E10+11, E10+12, E10+13, E+10+14 and E10+16 in ISS; and E10+19 in ISM (Fig. 2). They all alter the alternative splicing of exon 10 by either promoting or inhibiting exon 10 inclusion.

The exon-intron interface at the 3' end of exon 10 displays a high degree of self-complementarity, suggesting the presence of a stem loop (Fig. 2). Eleven mutations causing FTDP-17 are clustered in this stem loop region. They all disrupt the complementarity and destabilize the stem loop structure, leading this region of mRNA more available for association to U1 snRNP and resulting in exon 10 inclusion. In rodents, this stem-loop structure is destabilized by the replacement of a with g at position E10+13 (Fig. 2), which is also seen in FTDP-17 [35]. This replacement might explain why adult mice and rats express 4R-tau predominantly in their brains.

In addition to the regulatory sequences (*cis*-elements) within exon 10 and intron 10, distal exonic sequences appear to affect exon 10 splicing of tau as well. Disease-related mutations within exon 9 and exon 12 are reported to alter exon 10 splicing [37,38]. However, how and by which mechanism these distal sequences regulate exon 10 splicing remain to be elucidated.

### Regulation of *tau *exon 10 splicing by Trans-acting factors

Alternative splicing is highly regulated by *trans-*acting factors in addition to *cis*-elements. These splicing factors are divided into two major groups, hnRNPs (heterogeneous nuclear ribonucleoproteins) and SR (serine/arginine-rich)/SR-like proteins. Both of them are involved in alternative splicing [39,40]. SR/SR-like proteins are components of spliceosome. In addition, hnRNPs are also involved in pre-mRNA transport, RNA stability and translational regulation.

SR proteins are highly conserved in eukaryotes. They are characterized by containing one or two RNA-recognition motifs at the N-terminus, which determine RNA binding specificity, and an arginine-serine-rich (RS) domain at the C-terminus, which promotes protein-protein interactions within the splicing complex [41,42]. They are essential for both constitutive splicing and alternative splicing. For constitutive splicing, SR proteins are required for the formation of the early prespliceosomal complex to stabilized U1 snRNP article and U2AF [43,44]. In alternative splicing, SR proteins function in modulating 5' splice site in a concentration-dependent manner.

SF2/ASF (splicing factor 2/alternative splicing factor) is a well-studied SR protein. It binds to PPE enhancer of exon 10 (Fig. 2.) and plays essential and regulatory role in tau exon 10 splicing [45]. FTDP-17 mutations N279K and Δ280 K alter the normal PPE sequence by adding or removing an AAG copy and lead to increase or decrease in the binding ability of SF2/ASF, resulting in exon 10 inclusion and exclusion, respectively. In addition to SF2/ASF, roles of other SR proteins in exon 10 splicing are summarized in Table 1. All these studies were carried in cultured cells, and some observations are contradictory. Variations in the minigene size used, various types of cells with different compositions and levels of endogenous splicing factors as well as SR protein kinases and phosphatases, and different stages of cells may contribute to the inconsistent results among studies.

**Table 1:** Roles of SR/SR-like proteins in tau exon 10 splicing.

SR Protein	Target *cis*-element	Effect on exon 10 splicing	References
SRp20	ND	Exclusion	[68]
ASF (SRp30a)	PPE	Inclusion	[45,69]
SC35 (SRp30b)	SC35-like	Inclusion	[56]
SRp30c	ND	Inclusion	[69]
SRp40	ND	No Effect	[68]
9G8	ISS	Exclusion	[70]
SRp54 (SFRS11)	PPE	Exclusion	[71]
SRp55	ND	Exclusion	[68]
SRp75	ND	Exclusion	[72]
Tra2β	PPE	Inclusion	[73]

ND, not determined

### Phosphorylation of SR proteins

The RS domain of SR proteins is extensively phosphorylated on serine residues, and phosphorylation plays an important role in regulating their nuclear activities. To date, multiple kinases, including SR protein kinase 1 (SRPK1) [46], SRPK2 [47], cdc like kinase (Clk/Sty) [48], DNA topoisomerase I [49], cAMP-dependent protein kinase (PKA) and AKT [50,51], have been shown to phosphorylate the RS domain. Phosphorylation of the RS domain of ASF/SF2 promotes its interaction with pre-mRNA and other splicing factors and regulates the shuttling crossing nuclear membrane [48,52,53]. It has been shown that phosphorylation of ASF by SRPK1 drives it from cytosol into the nucleus and by Clk/Sty causes its release from speckles, the storage compartment of inactive SR proteins [52,54]. Thus, both SRPK1 and Clk/Sty help recruit ASF into nascent transcripts, resulting in enhancement of its role in regulation of alternative splicing. In the case of tau exon 10 splicing, activation of SRPK1 and Clk/Sty could increase the nuclear concentration of active ASF/SF2 that might result in an increase in exon 10 inclusion. Recently, we have found that dual-specificity tyrosine-phosphorylated and regulated kinase 1A (Dyrk1A), a critical kinase linked with DS, also phosphorylates ASF/SF2 at sites rather than those by SRPK and Clk/Sty and drives ASF/SF2 into speckles, resulting in suppression of its promotion in exon 10 inclusion (Shi J et al., unpublished observations).

The activity of SC35, which promotes tau exon inclusion, is regulated by phosphorylation with glycogen synthase kinase-3β (GSK-3β), a protein kinase that may be involved in the pathogenesis of AD [55]. Inhibition of GSK-3β activity in cultured neurons caused an increase in tau exon 10 inclusion [56]. However, the splicing competency of GSK-3β-phosphorylated SC35 in general or on tau is unknown. This issue is especially relevant because GSK-3β might be up-regulated in AD brain [57] and a SC35-like ESE at the 5' end of tau exon 10 appears essential for exon 10 splicing [35].

## Disruption of *tau *exon 10 splicing in tauopathies

Discovery of tau mutations in subjects with FTDP-17, a group of clinically heterogeneous syndromes with overlapping behavioral, cognitive and motor abnormalities, established that dysregulation of the *tau *gene or abnormalities of tau protein can trigger neurodegeneration [33,34,58]. In FTDP-17, at least 39 different mutations in the human *tau *gene have now been reported (Table 2). These mutations may be divided into two groups: (1) missense or deletion mutations that commonly modify tau interaction with microtubules, and (2) splicing mutations that affect the alternative splicing of exon 10, leading to changes of the ratio of 3R-tau/4R-tau. The 24 missense mutations are located in coding-region in exon 1 (R5H and R5L), exon 9 (K257T, I260V, L266V and G272V), exon 10 (N279K, N296H, P301L, P310S, G303V, S305N and S305I), exon 11 (L315R, L315L, S320F and S320Y), exon 12 (Q336R, V337M, E342V, S352V and K369I) and exon 13 (G389R and R406W). The two deletion mutations (Δ280K and Δ296N) are located in exon 10. The four silent mutations (L284L, N296N and S305S, L315L) are located in exons 10 and 11. There are eight intronic mutations in the splicing region of intron 10 (E10+3, E10+11, E10+12, E10+13, E10+14, E10+16, E10+19, E10+29) and one of intron 9 (E9+33).

**Table 2:** *Tau *mutations associated with FTDP-17

Mutation	Location	E10 inclusion	MT-binding	Insoluble tau	Phenotype
R5L	Exon 1			Mainly 4R	PSP-like
R5H R	Exon 1			4R+1N3	AD-like
K257T	Exon 9		↓	3R > 4R	PiD-like
I260V	Exon 9			Mainly 4R	
L266V	Exon 9	↓	↓	Mainly 3R	PiD-like
G272V	Exon 9	→	↓	Mainly 3R	PiD-like
E9+33	Intron 9	↓			
N279K	Exon 10	↑	Variable	Mainly 4R	PSP-like
Δ280K	Exon 10	↓	↓	3R>>4R	FTDP-17
L284L	Exon 10	↑	→	4R?	AD-like
N296N	Exon 10	↑	→	Mainly 4R	CBD-like
N296H	Exon 10	↑		Mainly 4R	FTDP-17
Δ296N	Exon 10		↓		PSP-like
P301L	Exon 10	→	↓	Mainly 4R	FTDP-17
P301S	Exon 10	↑		Mainly 4R	FTDP-17, CBD-like
G303V	Exon 10	↑		Mainly 4R	PSP-like
S305N	Exon 10	↑	→	Mainly 4R	CBD-like
S305S	Exon 10	↑		Mainly 4R	PSP-like
S305I	Exon 10	↑		Mainly 4R	AGD
E10+3	Intron 10	↑	→		FTDP-17
E10+11	Intron 10	↑	→		FTDP-17
E10+12	Intron 10	↑	→	Mainly 4R	FTDP-17
E10+13	Intron 10	↑	→		FTDP-17
E10+14	Intron 10	↑	→	Mainly 4R	FTDP-17, PSP-like
E10+16	Intron 10	↑	→	Mainly 4R	PSP/CBD-like
E10+19	Intron 10	↓	→		
E10+29	Intron 10	↓	→		
L315 R	Exon 11	→	↓		PiD-like
L315L	Exon 11		→		
S320F	Exon 11	→	↓		PiD-like
S320Y	Exon 11				PiD-like
Q336R	Exon 12	→	↑		PiD-like
V337M	Exon 12	→	↓		FTDP-17
E342V	Exon 12	↑		Mainly 4R	FTDP-17, PiD-like
S352V	Exon 12				
K369I	Exon 12			3R + 4R	PiD-like
G389R	Exon 13	→	↓	4R > 3R	PiD-like
R406W	Exon 13	→		3R + 4R	PSP-like

↑, increased; ↓, decreased; →, unchanged.

Majority of the missense and deletion mutations of tau also disrupt normal tau exon 10 splicing. The splicing mutations may cause FTDP-17 solely by disrupting the alternative splicing of exon 10 and consequently changing the ratio of 3R-tau/4R-Tau. Majority of the disease-causing tau mutations promote tau exon 10 inclusion, resulting in increase expression in 4R-tau (Table 2). However, there are a few mutations, such as L266V, G272V, Δ280K, E10+19 and E10+29, promote exon 10 exclusion and cause an increase expression in 3R-tau. Normally, adult human brain expresses approximately equal levels of 3R-tau and 4R-tau. Discovery of the splicing mutations in FTDP-17 demonstrates that disruption of 3R-tau/4R-tau balance is sufficient to causes neurodegeneration and dementia. A balanced 3R-tau/4R-tau ratio appears to be critical for maintaining normal brain functions.

In addition to FTDP-17, dysregulation of tau exon 10 splicing in both familiar and sporadic cases may also contribute to other human neurodegenerative disorders, such as PiD, PSP, and corticobasal degeneration. Some tau gene mutations can cause hereditary PiD and PSP [59-62]. Only 3R-tau inclusions were previously found in the brains of both familial and sporadic cases of PiD. However, several groups recently observed 4R-tau inclusions as well [63], suggesting that a disruption of 3R-tau/4R-tau ratio at either directions may contribute to PiD. Changes of 3R-tau/4R-tau ratio are also seen in PSP and corticobasal degeneration, in which 4R-tau is up-regulated in majority of the cases [63]. DS cases always develop tau pathology about 20 years earlier than sporadic AD. We recently found that the 3R-tau/4R-tau ratio increases in DS brain, suggesting that an imbalanced tau isoforms may also contribute to the early-onset tau pathology (Shi J et al., unpublished observations).

Altered ratio of 3R-tau/4R-tau was also reported in AD brain, but the observations from different reports are contradictory [64-66]. AD can be caused by multiple etiological factors. It is possible that there are several subtypes of AD, in which the 3R-tau/4R-tau ratio is differentially deregulated.

In FTDP-17, the altered tau exon 10 splicing is the result of tau mutations at the *cis*-elements that regulate the splicing, though the detailed mechanisms might be different in different mutations. Much less is known about the mechanisms by which the 3R-tau/4R-tau ratio is altered in other tauopathies. Further investigation on the mechanisms will help identify new therapeutic targets for the treatment of those tauopathies caused or contributed by disruptions of 3R-tau/4R-tau balance.

How the imbalance of 3R-tau/4R-tau causes or contributes to neurofibrillary degeneration and dementia is currently not understood. Since equal levels of 3R-tau and 4R-tau appear to be essential for normal function of the mature human brain, it is possible that the 1:1 ratio of 3R-tau/4R-tau bound to MTs is required for maintaining the normal dynamics of MTs in mature neurons. Because the MT-binding and MT assembly activity of 3R-tau is smaller than that of 4R-tau [23-26], any changes of the 3R-tau/4R-tau ratio could alter the MT dynamics and cause problems in the neuron. It is also possible that in the mature neuron, 3R-tau/4R-tau only at an 1:1 ratio bind to MTs. Access amounts of either 3R-tau or 4R-tau due to disrupted tau exon 10 splicing could resulted in increased concentration of free 3R-tau or 4R-tau in the cytoplasm. Compared to MT-bound tau, free tau is more vulnerable for hyperphosphorylation and aggregation into NFTs [67].

## Concluding Remarks

Tau is an important MT-associated protein in the neuron. *Tau *transcripts undergo alternative splicing of exons 2, 3 and 10, which produce six tau isoforms in the adult human brain. Alternative splicing of exon 10 is especially important because not only it determines whether 3 or 4 MT-binding repeats of tau are expressed, but also deregulation of this splicing causes or contributes to neurodegeneration and dementia. Regulation of *tau *exon 10 splicing is governed by at least 7 *cis*-elements located at exon 10 and intron 10 as well as many *trans*-acting splicing factors. Discovery of intronic mutations of *tau *gene in FTDP-17, which result in altered exon 10 splicing and neurodegeneration, had led to studies on the regulation of splicing at this site. To date, nearly two dozens of mutations of *tau *gene and one dozen of splicing factors have been shown to participate in regulation of *tau *exon 10 splicing. Nevertheless, the molecular mechanism of regulation of tau exon 10 splicing is still poorly understood.

Disruption of *tau *exon 10 splicing causes altered the expression ratio of 3R/4R-tau in several tauopathies. In FTDP-17, the altered 3R/4R-tau ratios are caused by mutations of *tau *gene. In other tauopathies such as PiD, PSP, corticobasal degeneration and DS, the exact mechanisms leading to the altered 3R/4R-tau ratios remain to be elucidated. The fact that the intronic tau mutations, which only disrupt tau exon 10 mutations but do not change the primary sequence of tau protein, result in FTDP-17 indicates that disruption of *tau *exon 10 splicing and/or altered 3R/4R-tau ratio are sufficient to induce neurodegeneration and dementia. Further understanding of the molecular mechanisms by which 3R/4R-tau ratio is disrupted and how the disruption induces neurodegeneration in some tauopathies will provide new insight into the mechanisms of these tauopathies and help identify new therapeutic targets to treat these disorders.

## Abbreviations

3R-tau: tau with three-microtubule-binding repeats; 4R-tau: tau with four-microtubule-binding repeats; ACE: A/C-rich enhancer; AD: Alzheimer disease; AGD: argyrophilic grain dementia; CBD: corticobasal degeneration; DS: Down syndrome; Dyrk1A: dual-specificity tyrosine-phosphorylated and regulated kinase 1A; ESE: exonic splicing enhancer; ESS: exonic splicing silencer; FTDP-17: frontotemporal dementia with Parkinsonism linked to chromosome 17; GSK-3β: glycogen synthase kinase-3β; ISE: intronic splicing enhancer; ISS: intronic splicing silencer; MT: microtubule; NFTs: neurofibrillary tangles; PHFs: paired helical filaments; PiD: Pick's disease; PPE: polypurine enhancer; PSP: progressive supranuclear palsy; RS: arginine-serine; snRNA: small nuclear RNA; snRNP: small nuclear ribonucleoprotein particles; SR: serine/arginine; SRPK: serine/arginine protein kinase.

## Competing interests

The authors declare that they have no competing interests.
